# Visual Hypo and Hypergnosia as Exemplars of Poles of Psychic Tonus in the Occipital Lobes: Multiple Case Analyses

**DOI:** 10.1155/2008/139436

**Published:** 2008-12-18

**Authors:** Claude M. J. Braun, Anik Guimond

**Affiliations:** Institut de Sciences Cognitives and Department of PsychologyUniversité du Québec à MontréalMontréalCanada

**Keywords:** Visual representation, hallucination, psychic tonus, hemispheric specialization, hypognosia

## Abstract

The “psychic tonus” model or PTM [1] of hemispheric specialization states that the left hemisphere is a psychic and behavioral activator and that the right hemisphere is an inhibitor. The PTM predicts that the tonus of visual representation ought to manifest hemispheric specialization in the occipital lobes. Specifically PTM predicts that pathological positive visual tonus (visual hallucination) ought to be associated more frequently with right occipital lesions. PTM also predicts that pathological negative visual tonus (loss of visual imagery) ought to result more often from left occipital lesions. We reviewed 78 cases of post lesion hallucination and 12 cases of post lesion loss of evocation of images, all following a unilateral lesion. Analyses of these relevant previously published cases support the predictions. In accordance with previously published demonstrations of hemispheric specialization for auditory tonus in the temporal lobes and for somesthetic tonus in the parietal lobes, the present findings extend the psychic tonus model of hemispheric specialization to vision in the occipital lobes.

## 1. Introduction

### *1.1. Psychic tonus: A hemispheric specialization interpretation of positive and negative symptoms*

Braun [1] has developed a model of hemispheric specialization principally based on effects of focal lesions which he has termed "psychic tonus". This model proposes a new framework for interpretation of positive and negative behavioral symptoms. The model draws equally from psychiatry as it does from neurology as well as biopsychology of normal function in humans and animals. The model is based on demonstrations to the effect that psychomotor baseline [2,3], libido [3,4], talkativeness [3,5], immune function [6], memory [7], audition [8] and somesthesia [9] are all modulated in similar opposed ways by the two hemispheres in right handers. The normal left hemisphere increases psychic tonus while the right decreases it.

The model predicts that post lesion visual anomalies should also manifest the same (opposite) dissociation according to lesion side. Left hemisphere lesions should produce negative visual anomalies (dulling of visual representation, i.e., of imagination) because of direct loss of the specialized circuitry of the damaged hemisphere. Right hemisphere lesions should produce positive visual anomalies such as hallucinations due to release of the specialized circuitry of the undamaged hemisphere. It is unusual but not unheard of [10] for dulling or loss of perceptual representations and hallucinations to be considered flip sides of a same coin. To make this point clear we will term positive distortion of perception "hypergnosia" and negative distortion "hypognosia". A mental image can be disturbed in two directions on a continuum. It can be over-excitable too

ISSN 0953-4180/08/$17.00 © 2008 – IOS Press and the authors. All rights reserved lush, too rich, too driven, hypertonic, i.e., *hypergnosic* on the one hand. However it can be under-excitable, frugal, impoverished, sluggish, hypotonic, i.e., *hypognosic* on the other. The former, in extreme form, is none other than visual hallucination. The latter, in extreme form, is none other than the loss of visual imagery (in the presence of intact reception sensation and elementary visual perception).

### *1.2. Disorders of visual perception*

Short term visual memory and imagination are barely distinguishable constructs. There have been many functional imaging investigations of encoding of visual material, verbal or non verbal. Regarding lateralization of the activations reported, there is a strong constant. The left hemisphere is practically always activated more than the right during encoding into memory (see Cabeza & Nyberg [11], for a meta-review). Perhaps not surprisingly, this has been many times reported for verbal material. More surprisingly, the same hemispheric asymmetry has consistently been reported for non verbal material as well [11]. Some of this asymmetry has been interpreted as an effect of semantic (verbal) processing which is thought to occur even when participants are encoding non verbal material [11]. Farah and colleagues [12] reviewed a series of case reports of loss of "visual imagery" (in both visual fields) and concluded that the trend was indicative of a higher frequency of left hemisphere lesions. Goldenberg and Artner [13] tested visual imagery and other functions in groups of patients with right and left posterior cerebral artery lesions. They concluded that left hemisphere lesioned patients indeed had poorer visual imagery than right hemisphere lesioned patients but that this was due to a loss of visual knowledge about the objects rather than a loss of imagery per se. The same explanation comes up in reviews of findings to the effect that left hemisphere lesions cause memory impairment, including of non verbal material, far more often than right hemisphere lesions (see Gillespie, Bowen and Foster [14] for a meta-analysis of the literature). However, that argument does not explain why the left hemisphere is found to contribute more to memory than the right in animals (see Braun [1], for a review of that literature). To sum up, functional imaging and the anatomoclinical method both support a strong left hemispheric specialization for visual imagination or encoding into memory. Research with animals demonstrates that this hemispheric asymmetry is not entirely due to verbal-semantic processing during encoding or during imagination.

Visual hallucination is rarer than auditory or somesthetic hallucination, and thus functional imaging studies of visual hallucination are less numerous than of hallucination in those other modalities. Only one study [15] has investigated visual hallucination in large groups of patients with good sets of hallucinatory incidents. Oishi and colleagues [15] compared two groups of patients with Parkinson's disease (PD), a subgroup with visual hallucinations ($N = 24$) and another without ($N = 41$) using rCBF. They found right fusiform hypometabolism in the hallucinating group. Nagano-Saito and colleagues [16] also compared a few PD patients with and without visual hallucinations. They found left frontal activation in the hallucinating cases. O'Brian and colleagues [17] studied hallucination in a few patients with Lewy body dementia (LBD) using SPECT. They reported a correlation between left cingulate and parietal hypometabolism and visual hallucinations. Imamura and colleagues [18] carried out a similar study and reported right parietal and temporal activation in the hallucinating cases, not significant when controlled for multiple comparisons. Mori and colleagues [19] reported a similar study as well, and found no evidence of lateralization of activations. In these latter three studies of LBD there seemed to be very few hallucinating cases, and very few hallucinatory incidents, suggesting to us that these results should not be judged informative for hemispheric specializations in the normal brain. Adachi and colleagues [20] studied visual hallucination in five patients with Charles Bonnet syndrome (cases without focal brain lesions). Importantly, SPECT measurements were obtained when patients were and were not hallucinating. Hallucinations were clearly related to activations, particularly in the temporal lobes, but these activations occurred equally often in either hemisphere. Shedlack and colleagues [21] found MRI stigmata in 12 elderly Charles Bonnet patients, but there was no evidence of any asymmetry of the stigmata. Braun and colleagues [22] reviewed single case reports of post lesion visual hallucination and found that of 55 cases due to a unilateral brain lesion 35 presented a right hemisphere lesion ($p < 0.05$). In that review the intrahemispheric location of the lesion was unconstrained and the field in which hallucinations were experienced was not noted. To sum up, the functional imaging and lesion literatures on visual hallucination are not yet very informative about occipital lateralization, but the most credible evidence available [15,22] suggests a source weakly lateralized to the left hemisphere.

### 1.3. Statement of purpose

This project proposes to optimize exploitation of multiple case analysis to test the idea according to which hemispherically specialized psychic tonus would be a good explanation of hypergnosia and hypognosia in the visual modality expressed exclusively in the occipital lobes. To this end carefully selected unilateral post-lesion cases were reviewed using a classification system appropriate for the inference test foreseen at outset.

## 2. Method

Cases sought here were 1) non paroxystic cases of visual hallucination (in one or both fields) due to a unilateral lesion involving the occipital lobe 2) non paroxystic cases of selective loss of visual imagery in both fields in the awake subject due to a unilateral lesion involving the occipital lobe. All cases manifesting any epileptic sign (seizures or twitching or drop attacks or EEG paroxysm or evidence of successful control of the hallucinations with anticonvulsants) were excluded from this study because hallucinations can come from an ipsilesional paroxysm even in presence of a massive lesion (see [23] for an excellent demonstration of the latter). Manifestation of the target symptom of both groups was an exclusion criterion. EEG findings, presence of aphasia, the field in which the hallucination occurred, the simple (unformed) versus complex (formed) nature of the hallucination, evidence of obvious episodic memory content in the hallucintation, patient age, gender and hand preference, lesion location and etiology of the lesion, were all noted systematically for each case, in view of control analyses.

Each of these data bases will be submitted to a same conservative non parametric inference test (two tailed $Chi^2$ test) of preponderance of right or left sided lesions. Right hemisphere lesions are expected to produce hypergnosia (hallucinations) and left hemisphere lesions are expected to produce hypognosia (loss of imagination). As a side issue, we were interested in indirectly testing the idea according to which perilesional (i.e., ipsilesional) hypermetabolism could explain unilateral hallucinations. Perilesional hypermetabolism has been reported in several cases of unilateral hallucination [24–26], including selectively during hallucination [27–29]. In such cases, hallucinations are occasionally suspected of being irritative and paroxystic in nature [27]. However, ipsilesional hypometabolism has been more frequently reported [30–35], including selectively during the hallucinations [36,37]. In such cases, hallucinations are not (or are less) suspected of resulting from paroxystic activity. Contralesional hypermetabolism has been reported in a case with visual hallucinations [38], but this seems to be an unusual finding. We reasoned that if the perilesional release account of post lesion hallucination in a single field is the best explanation, then the majority of cases of unilateral hallucination should manifest their hallucinations in the contralesional (usually partially anopic) field, as noted initially by Kolmel in his review of 16 patients [39]. Given as much, we were interested in determining whether a psychic tonus effect (i,e., a lesion side effect), in the cases with hallucination, would be limited to cases with bilateral hallucinations, with complex hallucinations or with hallucinations laden with content from episodic memory, all more apt to reflect higher order hemispheric specialization. Though we suppose that a hemispheric specialization effect ought to be due to contralesional release, we planned to test that notion, if possible, with EEG findings (i.e., ipsilesional slowing) and cases with SPECT imaging.

## 3. Results

### 3.1. Positive disorders of visual perception

Cases with post-lesion visual hallucination were sought by means of multitudes of medline and google scholar searches and cross referencing from related readings. For purposes of testing the psychic tonus model, cases with hallucinations in both visual fields are in principle more interesting than cases with hallucinations in a single field. Indeed to the extent that such a specific experience as hallucination can be conceived as a peculiar manifestation of unbridled mentation, i.e., elevated mental tonus, then the hallucination is all the more plausibly interpreted as such if it invades both visual fields – precluding its interpretation as a banal contralesional effect. Along the same line of reasoning, cases with complex hallucinations and/or hallucinated elements drawn from episodic memory (a hallucination of one's mother for example) are more relevant in principle for testing the psychic tonus model because these phenomena more readily reflect a "general" mental state than a small unformed hallucination (e.g., a spot of light) occurring in the anoptic field. Also, cases with comfirmed slowing of EEG over the lesion and by the same stroke rejection of the hypothesis of an irrita-

Table 1
Non-paroxystic visual hallucinations in both visual fields following a radiologically or surgically objectified unilateral occipital lobe lesion

| Age at onset gender and hand preference | Locus of the lesion | Lesion etiology | Type of hallucination and location in the visual field(s) * = the hallucinated image is from episodic memory | Clinical considerations | Reference |
|---|---|---|---|---|---|
| 71 Female H? | Right occipital | Infarct | Complex visual hallucinations* in both visual fields | No EEG, seizures or psychiatric comorbidity reported | [61] Michel & Troost |
| 73 Female H? | Right occipito-temporal | Infarct | Complex visual hallucinations* in both visual fields | No EEG, seizures or psychiatric comorbidity reported | [62] Meadows & Munro |
| 66 Female H? | Right occipital | Infarct | Complex visual hallucinations in both visual fields | No EEG or seizures reported, reactive depression, psychosis, paranoia and delusions | [63] Mancusa & Cole |
| 70 Female H? | Left occipital | Infarct | Complex visual hallucinations in both visual fields | No EEG, seizures or psychiatric comorbidity reported | [64] Lance |
| 75 Male H? | Left occipital | Infarct | Complex visual hallucinations* in both visual fields | No EEG, seizures or psychiatric comorbidity reported | [39] Kolmel |
| 72 Female RH | Right occipital | Infarct | Complex visual hallucinations, apparently in both visual fields | Four EEGs revealed no paroxysmal activity, only slowing over the lesion, no seizures were observed, pre-stroke psychiatric status was normal | [65] Peroutka et al. |
| 77 Male RH | Right occipital | Infarct | Complex visual hallucinations in both visual fields | No EEG, seizures or psychiatric comorbidity reported | [66] Dodd et al. |
| 58 Male H? | Right occipito-temporo-parietal | Cerebro-vascular accident | Complex visual hallucinations in both visual fields | Slowing of EEG on left, no mention of seizures or of previous psychiatric morbidity | [67] Medina et al. |
| 37 Male RH | Left occipito-temporal | Infarct | Complex visual hallucinations* in both visual fields agitation and aggressiveness | Slowing of EEG on left, agitation and aggressiveness | [67] Medina et al. |
| 42 Male H? | Left occipital | Abcess | Complex visual hallucinations* in both visual fields of people he had seen before | No EEG, seizures or psychiatric comorbidity mentioned | [68] Lunardi et al. |
| 66 Male H? | Right occipital | Abcess | Complex visual hallucination* of objects (apparently in both fields) | No EEG, seizures or psychiatric comorbidity mentioned | [69] Patterson et al. |
| 53 Male RH | Right occipito-parietal | Glioblastoma | Complex visual perseveration (palinopsia)* in both fields of objects seen over the previous half hour | No EEG, seizures or psychiatric comorbidity mentioned | [70] Maillot et al. |
| 79 Female H? | Right occipital | Intracranial heamatoma | Complex visual hallucinations in both visual fields | No EEG, seizures or psychiatric comorbidity mentioned | [71] Mukaetova-Ladinsk et al. |
| 47 Female H? | Right occipital | Infarct | Complex visual hallucinations in both visual fields | No EEG, seizures or psychiatric comorbidity mentioned | [72] Strandgaard et al. |
| 68 Male H? | Left occipital | Infarct | Complex visual hallucinations* in both visual fields (central vision) | No EEG, seizures or psychiatric comorbidity reported | [61] Michel & Troost |
| 66 Male H? | Right occipital | Glioblastoma | Complex visual perseveration (palinopsia)* in both visual fields of objects and people | Normal EEG, no psychiatric comorbidity reported | [73] Lazaro |

Table 1, continued

| Age at onset gender and hand preference | Locus of the lesion | Lesion etiology | Type of hallucination and location in the visual field(s) * = the hallucinated image is from episodic memory | Clinical considerations | Reference |
|---|---|---|---|---|---|
| 64 Male RH | Right occipito-temporal | Ischemia | Simple visual hallucinations (metamorphopsia) in both visual fields mild prosopagnosia | Slowing of EEG at posterior right scalp, no psychiatric comorbidity reported | [32] Hoksbergen et al. |
| 62 Male H? | Right occipito-parietal | Infarct | Simple vusual hallucinations “dazzle” in central fields | No EEG, seizures or psychiatric comorbidity mentioned | [74] Landis et al. |
| 20 Male H? | Left occipital | Penetrating wound | Simple visual hallucinations (polyopsia) in both visual fields | No EEG or seizures reported, personality and psychological tests failed to disclose significant neurotic trends | [75] Bender |
| 56 Male H? | Right occipital | Metastatic tumor | Simple visual perseveration (palinopsia)* in both visual fields | Normal EEG, no mention of seizures or of psychiatric comorbidity | [76] Estefania et al. |

tive paroxystic perilesional source of the hallucination are in principle more interesting for a test of the psychic tonus model than cases without EEG.

The ideal post lesion case for testing the psychic tonus model would have a clearly unilateral lesion limited to the occipital lobe (the visual cortex), only one symptom (visual hallucination), the hallucination would be complex, would contain elements from episodic memory, and would deploy in both visual fields, the patient would be right handed, he or she would present no signs of seizures only slowing over the lesion in EEG and no paroxysmal activity, and he or she would not be too young nor too old. It is needless to say that such perfect cases (for a test of the theoretical model under consideration) of course are rather rare. At any rate all these considerations are explicitly accounted for and regrouped accordingly in the next tables -as far as the information was available in each case report.

Of the 78 cases of visual hallucination following a unilateral occipital lesion 48 (62%) had a right hemisphere lesion and 30 (38%) had a left hemisphere lesion. A Chi$^2$ test of probability indicates that this difference is significant (Chi$^2 = 4.2, p < 0.043$ two tailed) if one were to suppose that each hemisphere is equally at risk for a lesion (see Tables 1, 2 and 3). These results support the psychic tonus model.

We believe left and right hemisphere lesions indeed have equiprobable chances of occuring and of being selected for single case reports (in the absence of selection for type of visual disturbance). Duval and her colleagues [40] assembled 725 cases using similar inclusion/exclusion criteria as here, except that they were not selected for any particular cognitive or psychiatric problem (an IQ test was required). This is the largest such data base we are aware of. In that data base there were 387 patients with left hemisphere lesions and 338 with right hemisphere lesions (binomial probability against equiprobability: $p$ = NS, two tailed). Thus it can basically be assumed that when unselected for memory disorder type, the two lesion sides may be considered theoretically equiprobable (or very close to that) when drawn from previously published reports.

### 3.2. Negative distortions of visual perception

Focal brain lesions can affect visual tonus negatively. The best example in the current story line would be a weakening of one’s ability to evoke a visual representation from immediate memory. This loss of internal visual evocation is none other than loss of the imaginative function, i.e., loss of imagination or “mind’s eye”. For purposes of replicability and clarity of our venture we consider a pure loss of visual evocation in the awake mind as an inability to retain in short term memory a visual stimulus which could be properly described and/or drawn in presence of the stimulus. This represents something like the opposite of visual hallucination. There are very few cases of this function being selectively destroyed by a focal unilateral lesion. Some cases present signs of associative agnosia (problems of verbal description of the object) or other aphasic signs or imperfect drawing of the object at copy.

We collected all previously published cases that we could find of selective loss of visual imagery due to a unilateral lesion, specifically, loss of the ability to non verbally evoke an image in immediate memory while

Table 2
Non-paroxystic visual hallucinations limited to a single visual field following a radiologically or surgically objectified unilateral occipital lobe lesion

| Age at onset gender and hand preference | Locus of the lesion | Lesion etiology | Type of hallucination and location in the visual field(s) * = the hallucinated image is from episodic memory | Clinical considerations | Reference |
|---|---|---|---|---|---|
| 45 Male H? | Left occipio-parietal | Meningioma | Complex long lasting hallucinations, including palinoptic* in the contralesional visual field | No EEG, seizures or psychiatric comorbidity mentioned | [77] Mooney et al. |
| 66 Male M? | Right occipital | Infarct | Complex visual hallucinations* in the contralesional visual field | No EEG, seizures or psychiatric comorbidity mentioned | [61] Michel & Troost |
| 66 Female RH | Right occipital | Infarct + atrophy right temporal | Complex visual hallucinations in the contralesional visual field | EEG slowing over the lesion, no psychiatric comorbidity reported | [39] Kolmel |
| 65 Female H? | Right occipital | Infarct | Complex visual hallucinations* in the contralesional visual field | No EEG, seizures or psychiatric comorbidity mentioned | [64] Lance |
| 53 Male H? | Right occipital | Infarct | Complex visual hallucinations* in the contralesional visual field | No EEG, seizures or psychiatric comorbidity mentioned | [64] Lance |
| 73 Female H? | Right occipital | Infarct | Complex visual hallucinations* in the contralesional visual field | No EEG, seizures or psychiatric comorbidity mentioned | [64] Lance |
| 72 Male H? | Right occipito-parietal | Infarct | Complex visual hallucinations in the contralesional visual field | Normal EEG, no seizures or psychiatric comorbidity reported | [64] Lance |
| 74 Female H? | Right occipito-parietal | Infarct | Complex visual hallucinations, including heutoscopic*, in the contralesional visual field | No EEG, seizures or psychiatric comorbidity mentioned | [39] Kolmel |
| 48 Male H? | Left occipital | Infarct | Complex visual hallucinations in the contralesional visual field | No EEG, seizures or psychiatric comorbidity mentioned | [64] Lance |
| 64 Male H? | Left occipital | Infarct | Complex visual hallucinations in the contralesional visual field | No EEG, seizures or psychiatric comorbidity mentioned | [78] Benson & Rennie |
| 56 Male H? | Left occipital | Infarct | Complex persistent visual hallucinations in the contralesional visual field | No EEG, seizures or psychiatric comorbidity mentioned | [79] Kasten et al. |
| 70 Female H? | Left occipital | Infarct | Complex visual hallucinations in the contralesional visual field | No EEG, seizures or psychiatric comorbidity mentioned | [64] Lance |
| 68 Male RH | Left occipito-parietal | Oligodendroglioma | Complex visual hallucinations in the contralesional visual field | No EEG, seizures or psychiatric comorbidity mentioned | [39] Kolmel |
| 50 Female RH | Left occipito-parietal | Resected glioblastoma multiform | Complex visual hallucinations in the contralesional field | No EEG or convulsion noted except once prior to resection, no psychiatric comorbidity reported | [80] Freiman et al. |
| 59 Female RH | Right occipital | Stroke | Complex visual hallucinations, including palinoptic*, in the contralesional field | Normal EEG (few independent sharp waves) no convulsions mentioned, no psychiatric comorbidity reported, right hypometabolism on SPECT | [35] Sun & Lin |
| 64 Female RH | Right occipital | Resected tumor adenocarcinoma | Complex visual hallucinations* in the contralesional field | Absence of seizures is insisted upon by the authors but no EEG was carried out, there were no hallucinations prior to resection, past history of anxiety and depression | [81] Faber & Johnson |
| 49 Male H? | Right occipito-parietal | Excised tumor | Complex hallucinations (persons, objects) in the contralesional visual field | EEG slowing over the lesion, no evidence of paroxysms, right hypermetabolism in SPECT | [26] Vogeley |

Table 2, continued

| Age at onset gender and hand preference | Locus of the lesion | Lesion etiology | Type of hallucination and location in the visual field(s) * = the hallucinated image is from episodic memory | Clinical considerations | Reference |
|---|---|---|---|---|---|
| 65 Male H? | Right occipital | Arterio venous malformation | Complex visual hallucinations in the contralesional field | EEG indicates no seizures, only supralesional slowing, no psychiatric comorbidity reported, SPECT indicated right hypometabolism during the hallucinations | [37] Inafuku et al. |
| 73 Female H? | Left occipito-temporal | Infarct | Complex visual hallucinations* in the contralesional field | No epileptic signs in EEG, phenytoin before and during hallucinations did not prevent them, left hypoperfusion in SPECT | [82] Waragai et al. |
| 76 Male RH | Right occipital | Infarct | Complex visual hallucinations in the contralesional field | No EEG or seizures mentioned, "no history of previous neurological or psychiatric disorders" | [83] Beniczky et al. |
| 72 Male RH | Right occipital | Infarct | Complex visual hallucinations* in the contralesional field | No EEG, seizures or psychiatric comorbidity mentioned | [84] Cogan |
| 84 Female H? | Right occipital | Meningioma | Complex visual hallucinations in the contralesional visual field | No EEG, seizures or psychiatric comorbidity mentioned | [85] Nagaratnam et al. |
| 84 Female | Right occipital | Stroke | Complex visual hallucinations in the contralesional field | No EEG, seizures or psychiatric comorbidity mentioned, right hypometabolism on SPECT | [30] Assadi et al. |
| 63 Male RH | Left occipital | Infarct | Complex visual hallucinations* in the contralesional field | No EEG, seizures or psychiatric comorbidity mentioned | [86] Cole |
| ? Male H? | Left occipital | Stroke | Complex visual hallucinations* in the contralesional visual field | No EEG, seizures mentioned, psychiatric tests were negative | [87] Anderson & Rizzo |
| 33 Male H? | Left occipital | Arteriovenous malfrmation | Complex visual hallucinations (palinoptic)* in the contralesional visual field | No EEG, seizures or psychiatric comorbidity mentioned | [88] Ritsema & Murphy |
| 70 Male H? | Right occipital | Infarct | Complex hallucinations in contralesional field | No evidence of paroxysms in EEG, no psychiatric comorbidity reported | [89] Kompf et al. |
| 50 male H? | Right occipital | Infarct | Complex hallucinations in contralesional field | No evidence of paroxysms in EEG, no psychiatric comorbidity reported | [89] Kompf et al. |
| 62 Male RH | Left occipital | Infarct | Complex visual hallucinations, including palinoptic* in the contralesional field | No evidence of paroxysms in EEG, no psychiatric comorbidity reported | [90] Huna-Baron & Kupersmith |
| 54 Male H? | Left occipital | Infarct | Complex visual hallucinations in the ipsilesionalvisual field | No EEG, seizures or psychiatric comorbidity mentioned | [91] Manford & Andermann |
| 79 Male RH | Left occipital | Infarct | Complex visual hallucinations in the ipsilesional visual field | Normal EEG including during hallucinations, "no personal or family history of psychiatric disease or substance abuse" | [87] Anderson & Rizzo |
| 82 Female RH | Left occipital | Infarct | Simple visual hallucinations (diplopia) in the contralesional field | No EEG, seizures or psychiatric comorbidity reported | [90] Huna-Baron & Kupersmith |

Table 2, continued

| Age at onset gender and hand preference | Locus of the lesion | Lesion etiology | Type of hallucination and location in the visual field(s) * = the hallucinated image is from episodic memory | Clinical considerations | Reference |
|---|---|---|---|---|---|
| 50 Male RH | Right occipital | Thrombosis | Simple hallucinations and complex visual perseveretion (palinopsia) of a few seconds in the contralesional visual field | No EEG, seizures or psychiatric comorbidity mentioned | [92] Ardila et al. |
| 64 Female H? | Left occipital | Infarct | Simple hallucinations in contralesional field | Normal EEG with slowing over the lesion, left occipital hypometabolism on SPECT, no psychiatric comorbidity reported | [31] Flint et al. |
| 37 Female H? | Right occipital | Metastatic carcinoma | Simple hallucinations in the contralesional field | No EEG, seizures or psychiatric comorbidity mentioned | [93] Johnson |
| 35 Male H? | Left occipital | Tuberculoma | Simple visual hallucinations (palinopsia) in the contralesional visual field | No EEG seizures or psychiatric comorbidity mentioned | [94] Werring & Marsden |
| 84 Female H? | Left occipitl | Ischemia | Simple long lasting hallucinations in the contralesional field | No EEG, seizures or psychiatric comorbidity mentioned | [24] Bosley et al. |
| ? Female H? | Left occipital | Etiology not specified | Simple hallucinations in the ipsilesional visual field | No EEG seizures or psychiatric comorbidity reported | [87] Anderson & Rizzo |
| 22 Male H? | Right occipital | Penetrating wound | Simple visual hallucinations (polyopia) in the ipsilesional field | No EEG seizures or psychiatric comorbidity reported | [75] Bender |

being able to describe and/or draw an object provided it is within visual reach. All the cases found had a left occipital lesion except the case of Riddoch [41] who had a left temporo-parietal lesion. As far as we could determine all these cases had lost visual evocation in both visual fields. See Table 4.

All 12 cases of Table 4 had a left hemisphere lesion. The two tailed binomial probability of this occurring by chance is $p < 0.0005$. The profile strongly supports the psychic tonus model.

Most of the authors of these reports on loss of visual imagery are aware of the preponderance of left hemisphere lesions in loss of mental imagery. Most of them interpret their patient's loss as a verbal-imaginal problem. They believe language, particulary its semantic aspect, contributes a "code" which consolidates images in memory [42]. They seem to hint that there must exist a posterior module of language dedicated to this purpose or that modules of imagery are disconnected from those of language [12] -thus explaining why their patient is not aphasic outright. Several authors demonstrated that the patient could draw on copy but not from immediate memory [12,41,43–45]. Farah and colleagues [12] tested visual evocation likely to be totally unmediated verbally or semantically (pointing to the color corresponding to that of an object presented before) and indeed found a deficit. Several investigations found a deficit of facial recognition (recognizing an unfamiliar face presented before) [12,46–48]. This particular mental operation is probably not mediated verbally or semantically. Several cases were not aphasic at all. Three cases complained of having stopped dreaming (the [12,46,49] cases). Despite this, commentators still clung to a verbal (more specifically semantic) mediation concept of mental images to explain the lesion locus and deficit of visual evocation of the patients in Table 4. Our psychic tonus model proposes a different perspective on these cases. Without denying that language may certainly contribute to evocability of mental images our model proposes that there is simply a paucity of mental representation associated with activation of the intact right hemisphere and there is an exuberance of the same representation associated with activation of the intact left hemisphere. This balance of hemispheric specializations affects language as much as any other aspect of mental life, explaining why post lesion overtalkativeness (hyperlalia) is radically associated with right hemisphere lesions [3, 50]. Thus left hemisphere lesions can be expected to reduce spontaneous expression and evocability of both language and mental imagery as well as any other cognitive content (except perhaps for contents for which the right hemisphere is radically specialized, but we think there is no such thing).

Since visual hallucination and loss of visual imagery are two opposites, comparison of the 78 cases of oc-

Table 3
Non-paroxystic visual hallucinations following a radiologically or surgically objectified unilateral occipital lobe lesion (location of the hallucinations in the visual fields unmentioned)

| Age at onset gender and hand preference | Locus of the lesion | Lesion etiology | Visual symptoms | Clinical considerations | Reference |
|---|---|---|---|---|---|
| 40 Male RH | Left occipito-parietal | Infarct | Complex visual hallucinations of objects and persons (visual field not specified) | No EEG, no signs of seizures, no psychiatric comorbidity mentioned | [95] Bhaskaran & Prabhu |
| 82 Male H? | Right occipital | Infarct | Complex visual hallucinations (visual field not specified) | No EEG, no signs of seizures, no psychiatric comorbidity mentioned | [96] Dejerine et al. |
| 78 Male H? | Left occipital | Infarct | Complex visual hallucinations (visual field not specified) | No EEG, no signs of seizures, no psychiatric comorbidity mentioned | [96] Dejerine et al. |
| 56 Male H? | Right occipital | Infarct | Complex visual hallucinations (location unspecified) | Normal EEG, no mention of seizures, no psychiatric comorbidity reported | [97] Vaphiades et al. |
| 46 Male H? | Right occipital | Infarct | Complex visual hallucinations* (location unspecified) | Normal EEG, no mention of seizures, no psychiatric comorbidity reported | [97] Vaphiades et al. |
| 46 Female H? | Right occipital | Infarct | Complex visual hallucinations (location unspecified) | Right slow focus in EEG, no mention of seizures, no psychiatric comorbidity reported | [97] Vaphiades et al. |
| 72 Male H? | Right occipito-temporal | Infarct | Complex visual hallucinations (location unspecified) | EEG slowing on left side, no mention of seizures, no psychiatric comorbidity reported | [97] Vaphiades et al. |
| 60 Male H? | Right occipital | Atheroma | Complex visual hallucinations (of objects and persons)* location unspecified | No EEG, seizures or psychiatric comorbidity mentioned | [93] Johnson |
| 32 Female H? | Right parieto-occiptal | Infarct | Complex visual hallucinations* (visual field not specified) | No EEG, seizures or psychiatric comorbidity mentioned | [98] Critchley |
| 67 Male RH | Left occipito-temporal | Hemorrhage | Complex visual hallucinations (location unspecified) | No EEG, seizures or psychiatric comorbidity mentioned | [99] Ott & Saver |
| 66 Female H? | Right occipito-parieto-temporal | Hemorrhage | Complex long lasting visual hallucinations, prosopagnosia, topographical agnosia | No EEG, seizures or psychiatric comorbidity mentioned | [100] Dutton |
| 71 Female H? | Left occipital | Infarct | Complex visual hallucinations (location unspecified) | Normal EEG, no mention of seizures, no psychiatric comorbidity reported | [97] Vaphiades et al. |
| 67 Female H? | Right occipital | Infarct | Complex visual hallucinations (location unspecified) | Normal EEG, no mention of seizures, no psychiatric comorbidity reported | [97] Vaphiades et al. |
| 62 Male H? | Right occipital | Infarct | Simple visual hallucinations (location unspecified) | No EEG, seizures or psychiatric comorbidity mentioned | [97] Vaphiades et al. |
| 46 Male H? | Right occipito-temporal | Infarct | Palinopsia, not further characterised (location unspecified) | No EEG, seizures or psychiatric comorbidity mentioned | [74] Landis et al. |
| 46 Male H? | Right occipito-temporal | Infarct | Visual hallucinations (not further characterized nor localized) | No EEG, seizures or psychiatric comorbidity mentioned | [93] Johnson |

Table 3, continued

| Age at onset gender and hand preference | Locus of the lesion | Lesion etiology | Visual symptoms | Clinical considerations | Reference |
|---|---|---|---|---|---|
| 35 Male H? | Right occipital | Ischemia | Visual hallucinations (nature not further specified, location unspecified) | No EEG, seizures or psychiatric comorbidity mentioned | [101] Kanzaki et al. |
| 42 Male H? | Right occipito-parietal | Cystic lesion (unspecified etiology) | Visual hallucinations palinopsia (location unspecified) | No mention of EEG, seizures or psychiatric comorbidity | [74] Landis et al. |

Table 4
Non paroxystic bilateral loss of visual imagery in the waking state following a unilateral occipital lesion

| Age at onset gender and hand preference | Locus of the lesion | Lesion etiology | Visual symptoms | Clinical considerations | Reference |
|---|---|---|---|---|---|
| 19 Female H? | Left occipital | Closed head injury | Loss of mental images, associative agnosia, no aphasia | No EEG paroxysms or psychiatric comorbidity mentioned | [48] Davidoff & Wilson |
| 51 Male H? | Left occipital | Closed head injury | Loss of mental images, semantic access agnosia, no aphasia | No EEG, paroxysms or psychiatric comorbidity mentioned | [45] Riddoch & Humphreys |
| 56 Male RH | Left temporo-occipital | Infarct | Loss of mental images, visuo-imaginal constructional apraxia, no aphasia | No EEG, paroxysms or psychiatric comorbidity mentioned | [42] Grossi et al. |
| 60 Male H? | Left occipital | Infarct | Loss of mental images, optic aphasia (partial loss of verbal account of mental images), aphasia limited to visual mediation | No EEG, paroxysms or psychiatric comorbidity mentioned | [102] Manning & Campbell |
| 83 Male RH | Left temporo-occipital | Infarct and hemorrhage | Loss of visual imagery, mild anomia without other signs of aphasia | No EEG, paroxysms or psychiatric comorbidity mentioned | [44] Goldenberg |
| 65 Male RH | Left occipito-parietal | Infarct | Loss of visual imagery, agraphia, Gerstmann syndrome, word finding difficulty | No EEG, paroxysms or psychiatric comorbidity mentioned | [103] Levine et al. |
| 79 Male H? | Left occipito-parietal | Infarct | Loss of visual imagery, dyslexia, anomia, ipsilesional neglect | No EEG, paroxysms or psychiatric comorbidity mentioned | [104] Cocchini et al. |
| 63 Male RH | Left temporo-occipital | Infarct | Loss of visual imagery, alexia and anomia for visually presented objects were the only aphasic signs, loss of dreaming | No EEG, paroxysms or psychiatric comorbidity mentioned | [46] Basso et al. |
| 64 Male RH | Left temporo-occipital | Infarct | Loss of visual imagery, no aphasia, loss of dreaming | EEG "mildly abnormal", no paroxysms or psychiatric comorbidity mentioned | [12] Farah et al. |
| 47 Male RH | Left temporo-occipital | Infarct | Loss of mental images, optic aphasia, loss of dreaming, no aphasia | No EEG, paroxysms or psychiatric comorbidity mentioned | [49] Pena-Casanova et al. |
| 35 Male RH | Left temporo-parieto-occipital | Resected meningioma | Loss of visual imagery, dysphasia | No EEG, paroxysms or psychiatric comorbidity mentioned | [43] Deleval et al. |
| 43 Female RH | Left parieto-occipital | Hemorrhage | Loss of mental images, visual associative agnosia, evolving to optic agnosia | No EEG, paroxysms or psychiatric comorbidity mentioned | [47] Benke |

cipital post lesion visual hallucination (all the cases of Table 1) with the 12 cases of occipital post lesion loss of visual imagery (all the cases of Table 4) – for lesion side appears perfectly meaningful. The double crossed dissociation predicted by the psychic tonus model is highly significant (Chi$^2 = 15.8$ $p < 0.0005$).

### 3.3. *Secondary control analyses* (*all the cases N = 90*)

At first glance the results reported above seem to be in total concordance with the hypothesis of the psychic tonus model. Furthermore, the stronger lateralization of the hypognosic group than the hypergnosic group is also in accord with the literature reviewed in the introduction. However several contaminating variables could challenge our main result.

### 3.4. *The locus of the lesion*

Visual hallucinations (including palinopsia) and the loss of mental imagery are more often the result of an occipital or temporal lobe lesion than lesions located elsewhere (see [51] for a review). In the current study, occipital damage was an inclusion criterion but damage elsewhere was not an exclusion criterion. It was therefore thought useful to verify whether a lobar difference in lesion extension could explain the main finding of a crossed interaction between lesion side and type of visual disorder (hypo/hypergnosia). The locus of the lesion was coded for each lobe (frontal, temporal, or parietal) separately because a combined analysis would have violated the rule of independence of the replicates and cases. We partialed out presence/absence of a lesion in each lobe as well as volume of the lesion (number of lobes lesioned) in partial correlation analysis between lesion side and type of visual disturbance. The correlation (interaction) remained significant in each case (Rp $> 0.41$, $p < 0.0005$).

### 3.5. *Presence of aphasic symptoms*

Presence of aphasic symptoms (anomia, dysphasia, aphasia, impaired oral comprehension) was of interest because of the well documented hemispheric specialization of the left hemisphere for language (see [52] for a review). Presence/absence of aphasic symptoms could compete with the psychic tonus model. Such an eventuality would require a caveat in our interpretation of our main findings, or at the very least, statistical partialing out of this variable's effect in our test of the main hypothesis. As we expected their were more cases with aphasic symptoms in the hypognosic group (5/12) than in the hypergnosic group (1/78) (Chi$^2 = 27.3$, $p < 0.0005$). However the crossed interaction between lesion side and type of visual disturbance remained robust after partialing out of presence/absence of aphasia (Rp $> 0.38$, $p < 0.0005$).

Several other variables were also tested for the eventuality of providing an alternative explanation for the finding of a crossed dissociation of left and right lesioned cases as a function of type of visual disturbance. For example psychiatric comorbidity (psychosis or mania) could be associated with visual hallucination [53] and not loss of imaginal evocation. A sampling bias of etiology of the lesion is important to consider because irritative pathophysiology (ex: tumor) could cause effects contrary to stable lesions such as CVAs. Another concern is that certain etiologies (ex: head trauma) comprise subtle diffuse damage thereby compromizing the "lesion side" inference. A sampling bias of hand writing preference would be important because a significant proportion of left handers have inverted hemispheric specialization (at least for language). Age is of concern because juveniles are less hemispherically specialized than adults and because the very elderly have diffuse brain atrophy and could thus present "noisy" lesion side characterization as well. A gender sampling bias is relevant to consider because in many respects women have less hemispheric specialization than men [54]. Date of publication is pertinent because diagnostic methods have improved over the decades, particularly due to the passage from CT to MRI scanning. However, the crossed interaction between lesion side and type of visual disturbance remained robust after partialing out of all these variables (Rp $> 0.40$, $p < 0.0005$). In fact, none the control variables mentioned above, was significantly related to the interaction between lesion side and type of visual disturbance.

### 3.6. *More specific secondary control analyses* (*hallucination cases only N = 78*)

### 3.7. *Perilesional release*

Occipitally lesioned cases often present with hemianopia. In the neurologic literature it is well known that visual hallucinations often occur in the defective visual field [55]. It is thus of interest to analyse the incidence of hallucinations in each visual field. Of the 39 cases with hallucinations in a single field, 36 hallucinated in the contralesional field and only 3 in the ipsilesional field (Chi$^2 = 27.9$, $p < 0.0005$) indicating strong (though indirect) support for the idea according to which the source of the hallucination is perilesional (ipsilesional) release. Bosley and colleagues [24] provide a particularly interesting description of perilesional hyperperfusion during unilateral visual hallucination: they illustrate a slim ring of hyperperfusion on SPECT all around the lesion. It is noteworthy that unilateral hallucination is nearly always contralesional whether the lesion be in the left or right occipital lobe.

### 3.8. Contralesional release

Perilesional release, we think, could be independent of hemispheric specialization. After all, it is observed as a highly localized phenomenon –close to the lesion, and it probably generates only unilateral hallucinations (although that remains to be determined). The psychic tonus model does not contest perilesional release. Rather, it proposes that there should be more hallucinating cases with right hemisphere lesions and it supposes that that preponderance would be due to contralesional release. What then, in the current data base would be the evidence for contralesional release? First, with regard to EEG, there are 12 cases out of the grand ensemble of 90 manifesting unilateral EEG slowing, and ten of the twelve cases had ipsilesional slowing. This is a profile compatible with contralesional release. In addition, in the cases with EEG (N = 24, overall), in whom we are more certain that the source of hallucination was not perilesional paroxystic irritation, there was a significant preponderance of right hemisphere cases (Chi$^2 = 6.0$, $p < 0.015$). Secondly, in the grand ensemble of 90 cases here, there are six cases with unilateral hypoperfusion on SPECT, all ipsilesional. This is also compatible with contralesional release. Of these six cases, four (67%) had a right hemisphere lesion (NS).

We propose that it would be worthwhile to further investigate both eventualities, of perilesional hypermetabolism and of ipsilesional hypometabolism, as a function of presence or absence of hallucination, in one or both visual fields. We expect it will be found that the two types of mediation of hallucination occur, and even that each can occur in a same patient. We also expect that perilesional hypermetabolism will be found to be more locally distributed (around the lesion) and that ipsilesional hypometabolism will be found to be more widespread, diffusing throughout most of the hemisphere.

### 3.9. Complexity of the hallucination

A visual hallucination can be either simple (seeing colored spots flickering lights, etc., N = 13/78 cases here) or complex (seeing a living scene, an animal, a person etc., N = 62/78 cases here). The complexity of hallucination, thus defined, was not significantly associated with lesion side. Whether the hallucinations occurred throughout both visual fields (N = 21/78) or were restricted to only one field (N = 39/78) was unrelated to lesion side. Finally, cases whose hallucination(s) included elements of episodic memory (elements highly specific to the patient's history) (N = 28/78) were not more likely to have right hemisphere lesions than the cases without episodic memory content (N = 47/78).

## 4. Discussion

The results of this investigation support the psychic tonus model. Unilateral occipital lesions causing a "positive" disturbance of visual representation (hypergnosia) are more often located in the right hemisphere. Unilateral occipital lesions causing "negative" disturbance of visual representation (hypognosia) are always located in the left hemisphere of right handers.

Why would a focal left occipital lesion impair a patient's visual representation of the entire visual field? Such an effect cannot be attributed to damage of primary sensory tracts. Primary visual representation has been mapped in many species and has always been found to be strictly contralateral and to present the same topography in each hemisphere in each occipital lobe, including in man [56]. Likewise, why would a right hemisphere lesion be more likely to provoke hallucinations in both visual fields? These are high order cognitive phenomena not explainable as direct neurosensory effects. We propose that the cerebral hemispheres influence higher level representation via psychic tonus regardless of the modality (motor, somatic, auditory, visual, immune, etc.). Psychic tonus may be only one among several sorts of hemispheric specialization, or it may eventually be made to fit into a more general and well integrated theory of hemispheric specialization. Hemispheric specialization for psychic tonus is a proposition that goes against the dominant neuroscience zeitgeist of today which is cognitivism. Psychic tonus is an unusual way of contemplating telencephalic and *a fortiori* cortical function. Unconscious and/or involuntary functions have more typically been conceived to depend on phylogenetically primitive brain tissue (e.g. the autonomic nervous system, the limbic system), and that might indeed be the case, but the lesion data indicate so far that psychic tonus is neocortically implemented in the human brain.

It could be thought that the main mediator of the post lesion "sticky switch" is one or several neurotransmitters. Three candidates are central norepinephrine, serotonin and dopamine (all known to modulate functions subsumed under psychic tonus). Robinson and colleagues [57,58] ligated the middle cerebral artery of rats. Assays of brain catecholamines revealed 30 percent reductions of norepinephrine in the injured and uninjured cortex and locus coeruleus and a 20 percent reduction of dopamine in the substantia nigra in the right lesioned rats who had also become hyperactive. In contrast rats with left middle cerebral artery ligations did not become hyperactive and did not show any significant change in catecholamines in any of the brain areas studied. Similar asymmetry occurs in serotonin concentrations as a function of stroke side in humans [59]. It remains to be determined whether the latter effect is hemispherically symmetrical or not (e.g., a PET ligand study). In fact however we believe that such a neurotransmitter-mediated mechanism is not the principal determinant of hemispheric specialization for psychic tonus effects reported here and elsewhere. Superficial cuts in rat cortex modulate psychic tonus without affecting neurotransmitter concentrations [1], and ictal visual hallucination results far more often from left foci [60]. This corroboration of hemispheric specialization for psychic tonus cannot be explained as a chronic and major change of neurotransmitter concentration in the brain after lesions. In other words, it is presently best explained as true hemispheric specialization, in the full sense.

## Acknowledgments

This research was made possible by a grant from the Fonds de Recherche en Santé du Québec (FRSQ).
